# Characterization and identification of extrachromosomal circular DNA in cholangiocarcinoma

**DOI:** 10.1371/journal.pone.0322173

**Published:** 2025-05-05

**Authors:** Zar Zar Win, Hasaya Dokduang, Siriyakorn Kulwong, Watcharin Loilome, Nisana Namwat, Jutarop Phetcharaburanin, Thidathip Wongsurawat, Piroon Jenjaroenpun, Poramate Klanrit, Arporn Wangwiwatsin

**Affiliations:** 1 Department of Biochemistry, Faculty of Medicine, Khon Kaen University, Khon Kaen, Thailand; 2 Cholangiocarcinoma Research Institute, Khon Kaen University, Khon Kaen, Thailand; 3 Faculty of Medicine, Mahasarakham University, Maha Sarakham, Thailand; 4 Department of Systems Biosciences and Computational Medicine, Faculty of Medicine, Khon Kaen University, Khon Kaen, Thailand; 5 Division of Medical Bioinformatics, Department of Research, Faculty of Medicine Siriraj Hospital, Mahidol University, Bangkok, Thailand; Instituto Nacional de Medicina Genomica, MEXICO

## Abstract

Extrachromosomal circular DNAs (eccDNAs) have gained attention as key players in cancer heterogeneity, potentially associated with elevated oncogene copy numbers in many cancers. While the presence of eccDNA in both normal and cancer cells is confirmed, its influence on gene-level alterations in cancer cells remains largely unexplored. This study delves into the genomic profiles of eccDNA in cholangiocarcinoma (CCA), an aggressive biliary tract cancer with extensive heterogeneity and diverse molecular alterations, using a modified long-read CircleSeq method. We reveal distinct eccDNA characteristics in CCA compared to non-tumor cells, focusing on genic components and chromosomal origins. Analysing read depth differences in oncogene-containing eccDNA; we identified potential eccDNA candidates that may be relevant for CCA biology. Subsequent bioinformatics analysis was performed using the established CReSIL tool, revealing distinct patterns of these oncogenes, particularly genes in the RAS/BRAF pathway, suggesting a potential functional role. These findings highlight the remarkable heterogeneity and diverse origins of eccDNA in CCA. This study establishes the first profiling of eccDNA in cholangiocarcinoma and paves the way for further investigation of its potential contribution to oncogene amplification and disease progression.

## Introduction

Cholangiocarcinoma (CCA) is an epithelial bile duct cancer originating from different anatomical locations within the biliary tree [1]. CCA is a slow-growing tumor that can quickly metastasize to distant sites due to their proximity to the lymphatic and blood capillaries of the liver [2]. The anatomical location of the tumor renders it difficult to diagnose and resect, with nonspecific symptoms in earlier stages. The worldwide incidence rate has been gradually increasing over the past few decades and it accounts for 10% to 25% of all hepatobiliary malignancies [3]. CCA remains a major public health burden in Southeast Asia, where it has a strong association with liver fluke *Opisthorchis viverrini* [4]. Multiple genetic alterations have been highlighted in the biliary tumors, and ongoing prospective studies are helping to stratify patients for diagnosis and therapy purposes [5]. Among this molecular characterization, mutations occur more frequently in cancer-associated genes such as TP53 (mutated in 44.4% of cases), KRAS (16.7%), and SMAD4 (16.7%) genes in Ov-linked CCA [6]. Mutations of oncogenes and tumor suppressor genes, as well as epigenetic modifications, were observed with profound heterogeneity in CCA via many signalling pathway [7]. However, the details of the molecular players that influence the heterogeneity and molecular characteristics of CCA remain under investigation.

Extrachromosomal circular DNAs (eccDNAs) refer to eukaryotic intracellular circular DNA elements that are derived from chromosomes. Their existence has been known for decades in healthy cells as well as in various cancers [8,9]. EccDNAs are emerging as key players in both normal and cancerous processes [9,10]. Recent advances in high-throughput DNA sequencing and bioinformatics algorithms have empowered researchers to uncover a wealth of new evidence linking eccDNAs to cancer progression. In healthy cells, eccDNAs might contribute to essential functions such as stress resistance and evolution [11], functional enhancement [12] and genome stability maintenance [13]. In cancer, given that eccDNAs are free of centromere and could distribute independently of chromosomal DNA, they could fuel the amplification of cancer-causing genes and promote tumor diversity [10,12]. What is more, eccDNAs have been found in circulation of cancer patients as well as in animal graft model for cancer [9]. This makes eccDNA a fascinating research target - whether they may hold clues for early cancer detection, better treatments, or predicting disease progression.

An in-depth investigation of eccDNAs in the context of cancer biology is an active research area. In terms of copy-number diversity, commonly amplified oncogenes in eccDNAs demonstrated the cell-to-cell variability in cell lines; EGFR in brain tumour cell line (GBM39); HER2 in breast cancer cell line (BT474) and DHFR in methotrexate-resistant HT-29 colon cancer cell line [10]. Research has consistently shown that eccDNAs harbor a higher proportion of introns and repetitive sequences compared to their chromosomal counterparts [9,14,15]. In blood samples, eccDNA sequences tend to be much smaller, with a notable portion being less than 5000 base pairs (bp) and a majority falling under 500 bp [9,14]. In contrast, eccDNA from cell lines exhibits greater variation in size, ranging from a mere 100 bp to several million base pairs (megabases) [8,9,15,16]. However, to our knowledge, information regarding eccDNAs in CCA has not yet been investigated. Further research could enhance our understanding of cancer heterogeneity and potentially reveal eccDNAs as valuable diagnostic markers for cholangiocarcinoma.

Using Oxford Nanopore Technology (ONT), we investigated a number of unique eccDNAs associated with CCA and characterized eccDNA features in CCA *vs.* non-tumor cholangiocyte cell lines. This study aims to deepen understanding of the relationship between eccDNAs and their potential roles in CCA and identify candidate eccDNA-related features for further characterization of molecular mechanisms in CCA pathogenesis. To our knowledge, this work present the first attempt in characterization of eccDNA in CCA using long-read sequencing. We envisage this work to provide a foundation for future investigation of eccDNAs as potential diagnostic biomarkers for precision medicine.

## Materials and methods

### Cultivation and cell harvesting

The human cholangiocarcinoma cells (KKU213A) were cultured in Dulbecco’s modified Eagle’s medium (DMEM) culture medium supplemented with 10% fetal bovine serum (FBS) (Gibco) and 100 units/mL penicillin, and 0.1 mg/mL streptomycin (Invitrogen) at 37 °C with 5% CO_2_ and maximum humidity. The immortalized non-tumor cholangiocyte cells (MMNK1) were cultured in and Ham’s F12 complete medium supplemented with 10% FBS (FBS; Gibco) in a humidified incubator with 5% CO_2_ at 37 °C. Cells were maintained at 70–90% confluence and passaged regularly. For experiments, cells were trypsinized to collect as a pellet. After trypsinization, the cells were resuspended in medium and pelleted before further processing by centrifugation at 800 x g for 5 min.

### Genomic DNA extraction

Cell pellets were lysed with Proteinase K, Buffer ATL and purified using the MagAttract HMW DNA Kit (Qiagen) to rupture plasma cell membranes before incubation at 56 °C for 16 h, 900 rpm. DNA concentration and purity were quantified by Qubit 3.0 Fluorometer (Invitrogen) and Nanodrop.

### Enrichment of eccDNA

eccDNA was purified and performed sequencing using Circle-Seq [8,17], with the following modifications:

### Removal of linear DNA

To selectively enrich for eccDNAs in our samples, enzymatic digestion of genomic DNA were employed using exonuclease V RecBCD (NEB, M0345S, USA). Additional fresh reaction buffer, ATP, and exonuclease V were added every 24h. After 48 h digestion, subsequent thermal inactivation of the exonuclease at 70 °C for 30 min was followed by purification of the remaining circular DNA using 1.8X QIASEQ BEADS (Qiagen, QIA-333923).

### Removal of mitochondrial DNA

Two enzymatic strategies for mitochondrial DNA (mtDNA) depletion in this study were used: MssI endonuclease treatment and a Cas9-based approach. For the MssI approach, the post-exonuclease V DNA was treated with MssI/PmeI endonuclease (Invitrogen, IVGN0244) following the Circle-Seq method to linearize mtDNA [8]. For the Cas9 endonuclease approach, Cas9 nuclease (NEB, M0386S) was used with two sgRNAs targeting MT-ND1 and MT-ND5 regions using the sgRNA sequences provided by Feng et al. [18] and synthesized by Synthego (USA), following the published protocol [18] with some modifications - the final concentration of Cas9 was optimized by doubling the concentration of Cas9 enzyme. Subsequently, linearized mtDNA from either MssI/Pmel or Cas9 method was further digested by Exo V enzyme for 24 h.

### Assessment of chromosomal and mitochondrial DNA removal

Quantitative PCR (qPCR) was used to assess the removal efficiency of linear chromosomal and mitochondrial DNA after the enzymatic digestion. Specific primers were used - β-globin gene (HBB) for linear chromosomal DNA [17]; and MT-CO1, MT-ND1, and MT-ND5 for mtDNA. The primers for qPCR targeting mtDNA were designed using CHOPCHOP software (https://chopchop.cbu.uib.no), taking into account the position of sgRNA targets. Primer sequences and expected amplicon sizes are listed in Table 1.

**Table 1:** Primers used for real-time PCR analysis.

Gene	Forward primer (5’-3’)	Reverse primer (5’-3’)	Product size (bp)
HBB	TATTGGTCTCCTTAAACCTGTCTTG	CTGACACAACTGTGTTCACTAGC	173
MT-CO1	GCCCACTTCCACTATGTCCT	GATTTTGGCGTAGGTTTGGTCT	114
MT-ND1	ATTCCTAATGCTTACCGAACGA	AAATAGGAGGCCTAGGTTGAGG	259
MT-ND5	CTCGCCTTAGCATGATTTATCC	GTGGAAGCGGATGAGTAAGAAG	243

### Rolling-circle amplification of eccDNAs

To amplify the amount of eccDNAs for downstream processes, the exoV-digested DNA samples were used for Phi29 DNA polymerase-based isothermal DNA amplification by GenomiPhi V2 kit (Cytiva 25-6600-32), following the manufacturer’s protocol.

### Debranching of RCA products

The RCA product was debranched using T7 endonuclease I (NEB, Cat. No. M0302S) in NEBuffer 2 to resolve branchpoints within RCA amplicons.

### DNA library preparation for Oxford Nanopore Sequencing

Debranched DNA samples were used for library preparation following the Native Barcoding Kit 24 V14 protocol (SQK-NBD114.24, Oxford Nanopore Technologies). After tagmentation and adapter attachment, pooled libraries were primed and loaded onto a MinION flow cell (R10.4.1). The data acquisition and initial base calling were performed using the MinKNOW tool (Oxford Nanopore Technologies).

### Read processing

Raw sequencing reads (FAST5 format) were obtained to generate FASTQ files. High accuracy basecalling, demultiplexing and adapter trimming were performed using guppy_barcoder and Porechop v0.2.4 [19] with the following parameters: *-c dna_r10.4.1_e8.2_400bps_hac.cfg --num_callers 2 --cpu_threads_per_caller 1 --min_qscore 7 --recursive*. Resulting filtered and adapter-trimmed FASTQ files were used for subsequent analyses, including genome mapping, eccDNA identification and annotation, and common motif identification.

### Read mapping and eccDNA analysis

The sequence data were mapped to the human reference genome (GRCh38) downloaded from the UCSC genome browser using minimap2 (v2.26) [20] and parsed to Bigwig format for visualization using deeptools [21] subcommand *bamCoverage* with the following parameters: *--bam file.bam -o file.bw --binSize 10 --normalizeUsing RPGC --extendReads*. To investigate variability between replicates and cell lines based on genomic mapping locations, deeptools [21] subcommand *multiBamSummary* and *plotPCA* were used to generate a.npz file and to visualize a principal component analysis (PCA) plot based on the.npz file. CReSIL tool [15] was employed to specifically capture sequencing reads originating from eccDNAs, and to provide genic and non-genic annotation on the eccDNAs.

CReSIL workflow includes (1) reference-based trimming, (2) region and linkage identification, and (3) eccDNA detection, assembly, and annotation. In the reference-based trimming step, sequencing reads were pre-processed to remove ghost sequences and aligned to the human reference genome GRCh38 using minimap2. CReSIL then categorized the reads into three types: breakpoint reads, circular template concatenation (CTC) reads, and normal reads. Unlike linear chromosomes, which align contiguously to the reference genome, eccDNA contains breakpoints reads which are a hallmark of eccDNAs and indicate circularization. CReSIL detects these structural features by categorizing reads, preserving aligned blocks with specific strand orientations, and constructing graphical representations to confirm circularity. The identification of CTCs is particularly crucial in distinguishing eccDNAs, as they represent evidence of circularization events. Reads containing CTCs were trimmed to retain only the segments aligning with the reference genome in the correct orientation, while non-CTC reads that are characteristics of eccDNAs and cannot be detected in linear DNA were processed based on their alignment patterns.

In the second step which is region and linkage identification, BEDTools was used to merge chromosomal aligned regions, allowing the identification of potential eccDNA origins. Breakpoint reads were analyzed to establish linkages between regions, enabling the detection of circular structures. The finalized step was eccDNA detection, assembly, and annotation. To confirm eccDNA presence, CReSIL constructed a graph-based representation, mapping genomic regions as nodes and their linkages as edges. The tool applied a reference-guided assembly algorithm to extract and refine eccDNA sequences. Finally, genomic features—including exons, introns, CpG islands, and repeats—were annotated using the UCSC genomic database version GRCh38. Circos-compatible input files were generated to facilitate visualization of the identified eccDNAs and their genomic context.

Motif identification was performed using MEME [22] for 20 bp motif and TOMTOM [22]against HOCOMOCO v12 CORE database (human+mouse orthologs) [23]. Annotated genes from CReSIL output were used for pathway enrichment analysis on Reactome (https://reactome.org/). Downstream analysis, investigation and data visualization was performed using IGV [24], R (www.R-project.org), and Cytoscape (https://cytoscape.org/)

## Results

### Chromosomal origins of eccDNAs and heterogeneity between CCA and non-tumor cell lines

We adapted the Circle-Seq method [8,17] for eccDNAs purification from both CCA cell line (KKU213A) and MMNK1 cell line. A total of 12 samples were sequenced; DNA were extracted from two cell lines each prepared using two enzymatic methods (Cas9 or MssI) with 3 replicates per method followed by Oxford Nanopore sequencing. The sequencing yielded a total of 6.57 million reads for all samples. After applying quality filtering with a threshold cut-off of 10 and trimming adapter sequences, the processing resulted in an average of 255,228 high-quality reads being retained per sample (S1 Table). When compared in terms of methods, Cas9-based samples generated a higher number of reads than MssI treated samples (S1 Fig). The read length spanned between 200–86,226 base pairs. The GC content was slightly variable amongst the samples. The lowest GC content (36.9%) was observed in Cas9_KKU213A, replicate 1 and the maximum (40.7%) was observed in MssI_KKU213A, replicate 1 (S1 Fig). In terms of genomics and mtDNA removal, the qPCR results (S2 Table) and mapping region of HBB gene (S2 Fig) revealed complete removal of linear chromosomal DNA. While all methods left some mtDNA, the Cas9 approach appeared to be less efficient (S2 Fig, S2 Table). MssI achieved greater mtDNA removal, but concerns exist regarding potential off-target effects on eccDNAs as previously mentioned in prior research [14,18].

To investigate the unique eccDNAs, the pre-processed reads were analyzed using CReSIL to reconstruct circular DNA sequences. More than thousands of eccDNAs were identified from both cell lines (S1 Fig). MssI-treated KKU213A replicate 2 shows the highest number with 4,161 unique eccDNAs, while MssI-treated KKU213A replicate 3 exhibited the lowest number with 140 of unique eccDNAs. The count of eccDNAs can vary from one cell line to another [25]; however, our data has shown that the tumor samples generated a less consistent number of unique eccDNA than the non-tumor samples (S1 Fig).

**Fig 1:**
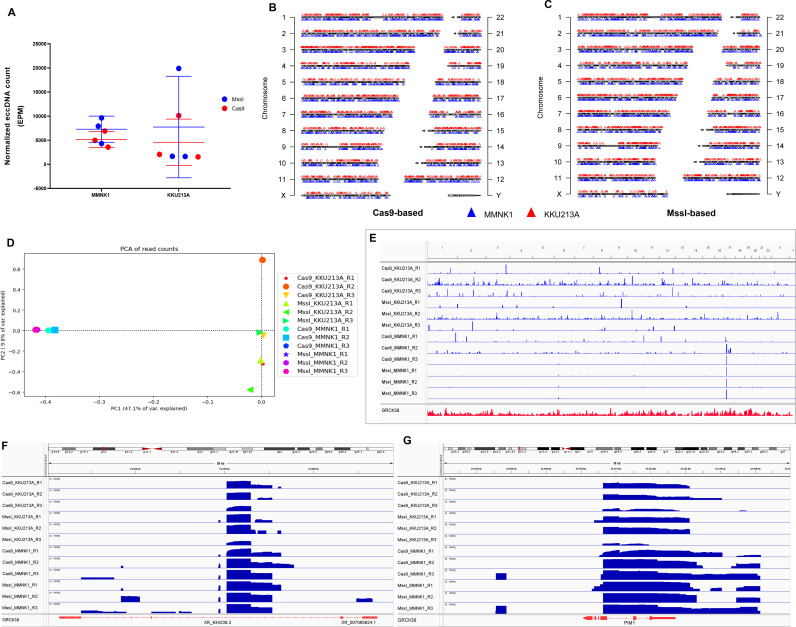
Genome mapping comparison. **(A)** Normalized eccDNA count per million of all samples. **(B and C)** Chromosomal distribution of eccDNAs origins in **(B)** Cas9-treated samples and **(C)** MssI-treated sample. **(D)** Principal component analysis of genomic coverage of all samples based on their mapped reads to the human reference genome. **(E)** Read depth distribution of all samples across the reference genome. **(F)** Region of lncRNA gene XR_934236.3 on chromosome 17. **(G)** Region of proto-oncogene PIM1 on chromosome 6.

To examine which method can offer a better enrichment of eccDNA, we analyzed the normalized eccDNA count per million reads across all samples. Our results revealed no significant differences between methods, prompting us to enrich our analysis by employing data both approaches instead of favoring a singular method (Fig 1A). Mapping revealed that eccDNAs can originate from any chromosome (Fig 1B, 1C), with chromosomes 3, 12, and 20 appeared enriched in CCA samples, and chromosomes 6, 7, and 12 in non-tumor samples (S3 Table). Hence, chromosome 12 was frequent in both cell lines. In addition, we found that the majority of eccDNAs contained regions from a single chromosome (simple form eccDNA). A small percentage was composed of regions originated from multiple chromosomes (complex form eccDNAs) (S1 Fig). EccDNAs from CCA samples showed slightly higher proportion of eccDNAs derived from multiple chromosomal fragments (S1 Fig).

To investigate the differences in eccDNA distribution patterns between CCA and non-CCA cell lines, principal component analysis (PCA) on the genome mapping locations obtained from the sequencing data were analyzed (Fig 1D). The result showed non-tumor samples clearly clustered away from the tumor samples, indicating distinct chromosomal origins. Additionally, non-tumor samples showed less variability, whereas the cancer replicates were more dispersed across the PC2 axis (Fig 1D). Exploring the mapped regions in more detail, read depth distributions and other features across the reference genome were visualized using IGV. Consistent with the PCA, the mapped locations of the tumor samples were less consistent across replicates compared with the non-tumor samples (Fig 1E). Interestingly, the data from tumor samples revealed markedly more dispersed read depth and genomic origins than the non-tumor ones, highlighting inter-sample variations in genomic coverage for the tumor cell line (Fig 1E).

In addition, we found a remarkable uniformity of read depth in some genomic area, regardless of nature of the samples. In particular, all samples from KKU213A and MMNK1 cell lines shared the same pattern on chromosome 17 (Fig 1F). In chromosome 17, all samples showed coverage in the region where lncRNA gene (XR_934236.3) occupied. Moreover, the reads from non-tumor samples covered a distinctly wider area of the gene where this uncharacterized *Homo sapiens* non-coding RNA transcript (XR_934236.3) exists Fig 1F. Similar pattern was observed on chromosome 6 where a proto-oncogene PIM1 is located (Fig 1G).

### EccDNA landscape: sizes and sequences of identified eccDNAs

**Fig 2:**
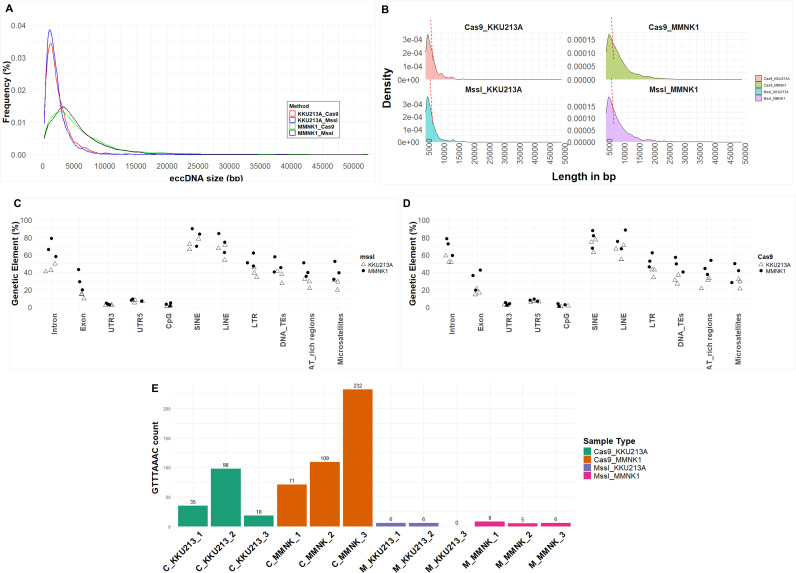
eccDNA characteristics in CCA. **(A)** The occurrence of eccDNAs under 3 kb in size was higher in tumor samples than in non-tumor samples. **(B)** Density plot of length distribution of eccDNAs across the samples, variability and peaks of eccDNA lengths were shown in the figure. **(C)** Genomic composition of identified eccDNAs in MssI-approach **(D)** Genomic composition analysis in Cas-9 approach. **(E)** The number of eccDNAs containing GTTTAAAC sequence, the recognition site for MssI enzyme.

The reconstructed eccDNAs from CReSIL [15] provided information on their characteristics including the length of each eccDNA, read coverage for each eccDNA, as well as the annotations with genomic contents. In terms of size distribution, all identified eccDNAs revealed considerably greater variation in length within tumor samples compared to non-tumor samples, supporting the potential roles of eccDNA length instability in tumor development. Moreover, tumor samples exhibited a greater enrichment of eccDNAs smaller than 3 kb compared to non-tumor ones (Fig 2A). MMNK1 appeared to possess eccDNAs that were longer than the KKU213A, regardless of the enzyme treatment (Fig 2B). There was an extensive variability in the length of eccDNAs, spanning between 300–7000 bp in KKU213A samples and 300–8000 bp in MMNK1 samples (Fig 2B). These distinct length distributions suggest possible differences in the nature and origin of eccDNAs between CCA and non-tumor cells.

### A Deep dive into eccDNAs: identifying genic and intergenic genomic elements

Characterization of genomic contents harbored by eccDNA is essential for understanding the phenotypic outcome of eccDNA in many diseases, particularly in the mechanisms by which eccDNAs may be involved in cancer. We examined the genomic composition of the identified and annotated eccDNAs. The analysis revealed a higher proportion of introns compared to exons across all samples irrespective of the approach (Fig 2C, 2D). Our finding aligns with previously published research [9,15]. Interestingly, non-tumor samples seem to be “richer” in genetic elements, harboring more introns and exons than the tumor counterpart (Fig 2C, 2D). For other non-genic contents, non-tumor samples possessed a higher number of genomic elements than in tumor ones most of the time (Fig 2C, D). SINE elements were the most prevalent repetitive elements found within eccDNAs, followed by LINE elements. The predominant presence of LINE and SINE elements in eccDNAs suggests potential roles in their stability, mobility, or function and could be involved in the regulation of gene expression [26].

Moreover, we explored the difference in MssI cleavage site abundance across sample groups. Understanding the distribution of these sites may predict the potential impact of MssI treatment on eccDNA enrichment and the observed size distribution patterns. In particular, the digestion of mtDNAs by MssI endonuclease, which recognizes a short nucleotide sequence of 5’ GTTTAAAC 3’ could affect some eccDNAs that harbored the same cutting sites, or the enzyme may have “star activity”. Hence, we identified the distribution of MssI recognized sequences and detected that MssI-treated samples clearly contained a smaller number of MssI cleavage sites than Cas9-based samples (Fig 2E). This demonstrated the possibility that some eccDNAs may be subjected to removal by the use of MssI enzymes. Arguably, they could be leftover mtDNA that were not cut by the sgRNA-cas-9 reactions; however, upon investigation of the chromosomal origins of these GTTTAAAC-containing eccDNAs, none of them were originated from mitochondrial chromosome.

### Motif discovery reveals sequence enrichment in tumor-derived eccDNAs

**Fig 3:**
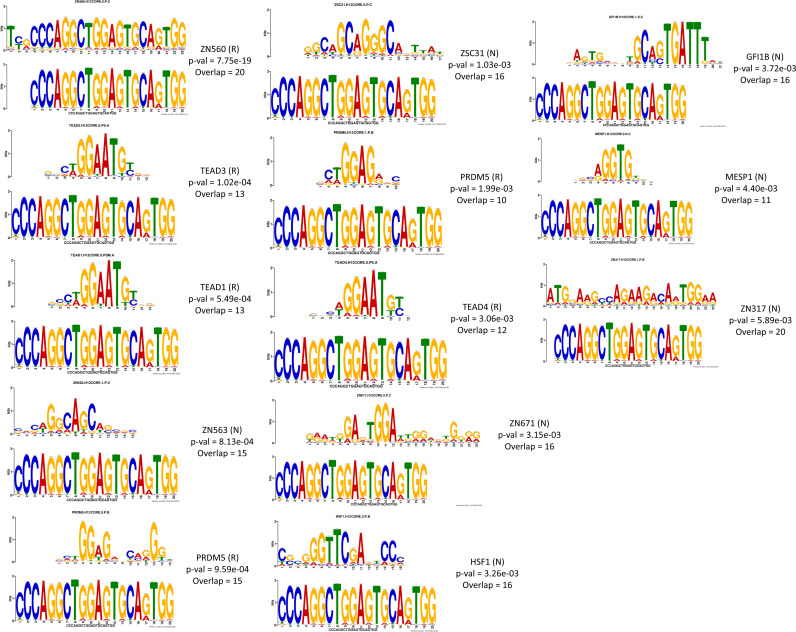
Recurring motifs in CCA-derived eccDNAs. Matches of the common eccDNA motif of KKU213A cell line found in HOCOMOCO v12 CORE database. P-value refers to the TomTom estimate value using a null model consisting of sampling motif columns from all the columns in the set of target motifs. (R = Reverse complement, N = Normal).

To identify potential regulatory motifs associated with eccDNAs, we employed the MEME tool [22] on the consensus sequences of eccDNAs. We configured the parameters with a motif length of 20 bp and sought the top 100 motifs within input FASTA sequences of Cas9-cleaved, MssI-digested, and pooled eccDNA-sequences for each cell type (both Cas9 and MssI combined). Interestingly, a single, recurring motif CCCAGGCTGGAGTGCAGTGG emerged only in tumor-derived eccDNA sequences, and no shared motif was observed in the non-tumor ones. Further functional analysis of all CCA recurring motif using TomTom [27] identified 13 motifs matching entries, including 4 kinds of C2H2-zinc finger factors and 3 types of TEAD domain transcription factors. Besides, the top motif revealed a significant match (p-value = 7.75e-19) between this 20 bp motif (Fig 3) and the known ZNF560 motif of the HOCOMOCO v12 CORE database (human+mouse orthologs) [23].

### Pathway network analysis of genes harboured by eccDNAs

**Fig 4:**
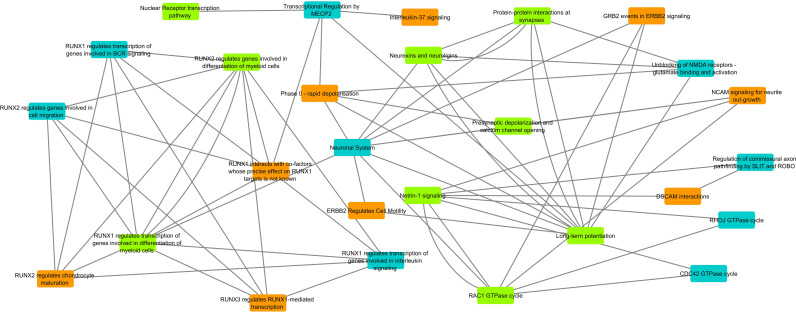
Pathway network analysis based on genes annotated in eccDNAs. Annotated genes in eccDNAs were used for pathway enrichment analysis. Each enriched pathway was represented as a node. Connected nodes indicated that they shared at least one gene from enrichment analysis. (Yellow = contained only CCA-derived genes, Blue = contained only MMNK1-derived genes, Green = contained both CCA and MMNK1 derived genes).

To evaluate potential roles of the genic contents in eccDNAs, the gene list from annotated eccDNAs for KKU213A or MMNK1 were used for pathway enrichment analysis using Reactome database. Cytoscape [28] was used to visualize the enriched pathways, connecting pathways that contain shared genes. Despite the fact that eccDNAs did not always contain full genes, the analysis displayed that CCA-derived eccDNA genes and MMNK1-derived eccDNA genes were observed in 9 unique pathways; and eccDNAs of both cell lines shared genes in 9 common pathways (Fig 4). RUNX driven pathways and multiple neurological-related pathways were observed in the analysis. Interestingly, all four genes (DSCAM, DCC, NTN1, DSCAML1) involved in DSCAM interaction pathways were observed only in CCA-derived data.

### Investigation of oncogenes on eccDNAs and their coverage level

Many studies have highlighted the amplification of oncogenes within eccDNAs across various cancer types, potentially contributing to enhanced expression of genes associated with tumorigenesis [10,12,14]. In this study, we identified concordant evidence for the association between the presence of oncogene-harbouring eccDNAs and the coverage of the eccDNAs.

To identify genes related to CCA, a total of 71 articles related to oncogenes relevant to CCA published up to date were retrieved from PubMed and ScienceDirect using search terms “CCA”, “Cholangiocarcinoma”, “KKU, Cholangiocarcinoma, Overexpression”. Through the initial screening of the abstract, 16 articles were excluded from the analysis as they did not fulfill the inclusion criteria: 1) studies of which CCA are the main subject 2) studies on oncogenes related to CCA 3) *in vitro*, *in vivo* in animal models, and 4) published as full articles. The remaining 53 articles were downloaded for full text assessment. Two articles were excluded from the analysis as they did not study oncogenes in CCA. Finally, a total of 51 articles had none of the exclusion criteria: 1) articles in languages other than English 2) articles on other cancers with inclusion of CCA 3) review articles and 4) studies not related to genes. We performed a manual search of relevant articles that include the most frequently found oncogenes in CCA regardless of their sub-anatomical location.

**Fig 5:**
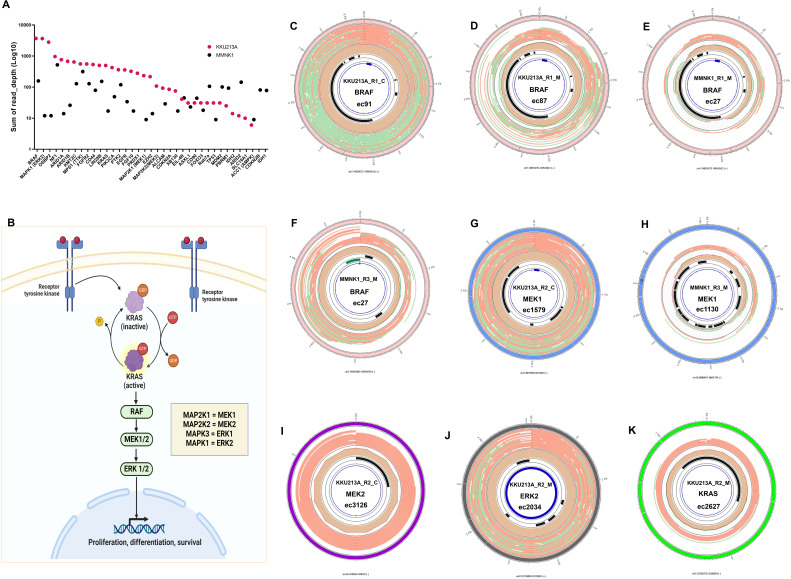
Analysis of CCA-associated genes and pathways in CCA and non-tumor cell lines. **(A)** Read depth of oncogene; the read depth values shown are summation of all 3 replicates for CCA and non-tumor cholangiocyte cell lines. Multiple genes, particularly those in the Ras/BRAF pathway showed elevated read depth in CCA cell line. **(B)** Illustration of RAS/BRAF Pathway: This schematic depicts the RAS-BRAF- MAP2K1-MAPK1 signaling pathway, highlighting key components and their interactions. **(C-K) Coverage of eccDNA carrying genes in RAF/BRAF pathway between CCA and non-tumor cholangiocyte cell lines: (C)**, **(D)** show high-coverage of BRAF-eccDNA in two samples of KKU213A (CCA cell line). **(E)**, **(F)** Low-Coverage of BRAF-eccDNA in two samples of MMNK1 (non-tumor cholangiocyte cell line). **(G)** High coverage of eccDNA carrying MAP2K1 (MEK1) gene in the KKU213A cell line. **(H)** Low coverage of eccDNA carrying MAP2K1 (MEK2) gene in the MMNK1 cell line. Additional genes found in eccDNAs linked to the RAF/BRAF pathway (MAP2K1 (MEK2), MAPK1 (ERK2) and KRAS) and these eccDNA-derived genes were only found in CCA samples **(I)** MAP2K1 (MEK2), **(J)** MAPK1 (ERK2) **(K)** KRAS. (C: Cas9-treated. M: MssI-treated. R: Replicate number. Blue region: BRAF gene. Black regions: repetitive elements. Green region: CpG-rich area).

Based on the literature review, the frequently harbored oncogenes in intrahepatic CCA were explored in more details from our eccDNA data. We investigated the frequently harbored oncogenes from our genome mapping data and from the coverage of eccDNAs generated from the CReSIL pipeline. The tumor-derived eccDNAs stood out with dramatically higher coverage on eccDNAs and mapping read depth across most oncogene regions (Fig 5A). Particularly striking was the prominence of the BRAF gene, which displayed markedly elevated read depth levels within tumor samples, far surpassing those observed in non-tumor cholangiocytes (Fig 5A). Both eccDNA coverage and genome mapping read depth pointed towards elevated levels in genes associated with the RAS/BRAF pathway (Fig 5B) and the OSBP2 gene in tumor samples (Fig 5A). This may indicate enhanced activity or amplifications of these genes in CCA, which might hold promise as potential biomarkers or therapeutic targets.

BRAF gene that localized on eccDNAs show predominantly higher coverage in KKU213A samples (Fig 5C, 5D) compared to MNNK1 samples (Fig 5E, 5F). Replicate variability requires further exploration, but the observed differences in coverage are noteworthy. In addition, comparison of eccDNAs read depth between the MssI and Cas9 approaches revealed distinct eccDNA enrichment patterns. For instance, certain oncogenes, such as BRAF, showed higher read depth when analyzed using Cas9 compared to MssI (Fig 5C-D) even in the same replicate of the same cell line, indicating differences in enzyme efficiency and specificity. What is more, MEK1 gene was also present at higher coverage in the KKU213A sample, contrasting with its small coverage in MMNK1 (Fig 5G, 5H). Further investigation of genes in the RAS/BRAF pathway also revealed the exclusive presence of KRAS, MAP2K1 (MEK2), and MAPK1 only in CCA-derived eccDNAs (Fig 5I, 5J, 5K). Notably, these identified genes are well-known players in this highly relevant pathway for CCA carcinogenesis, suggesting a potential role of eccDNAs and CCA development processes. Future investigation is warranted to explore the functional significance of these observations and their potential role in CCA pathogenesis.

## Discussion

To overcome the challenge of limited eccDNA quantity in cells, we optimized a multi-step enrichment protocol using two mitochondrial DNA removal methods followed by Phi29 DNA polymerase amplification. This approach successfully generated sufficient copies of eccDNAs from KKU213A (CCA) and MNNK1 (non-tumor cholangiocyte) cell lines for subsequent Oxford Nanopore sequencing. Our analysis indicated that MssI removed more mtDNA but potentially included off-target eccDNA fragments. Moreover, the read depth analysis in IGV indicated that MssI-treated samples showed lower coverage throughout the whole region, especially in non-tumour samples. These finding agree with previously published work [29] and this suggests that MssI-treated samples could lose large eccDNAs which hold a higher probability of carrying MssI restriction sites thereby affecting the profiles of eccDNAs. The sgRNA-Cas9 method might be precise for mtDNA depletion but not entirely effective. Feng et al. [18] reported 85.9% ± 12.6% efficiency for the sgRNA-Cas9 approach, citing obstacles like enzyme-substrate dynamics, SNP variations, and spacer design issues. Other factors affecting efficacy include mitochondrial heteroplasmy in cancer [30], RNA-DNA duplex stability, chromatin environment, and sgRNA loading [31].

To validate the specificity of our eccDNA isolation process, we assessed the removal of linear chromosomal DNA using qRT-PCR and visualized the results with IGV (S2 Fig). We selected the HBB gene as a representative chromosomal marker. The absence of detectable HBB signals in our eccDNA-enriched samples confirmed the that the linear chromosomal DNA was effectively removed, ensuring that our pipeline selectively captured circular DNA molecules (S2 Fig). High-throughput long-read sequencing revealed tens of thousands of eccDNAs in both tumor and non-tumor cell lines. Most eccDNA features (genic and non-genic) displayed were consistent with previous reports [8,9,14,15,29]. EccDNAs from our non-tumor samples exhibited longer lengths compared to those from the tumor samples. This observed length heterogeneity aligns with previously described eccDNA characteristics [8,9,14,29], potentially reflecting their unpredictable biogenesis [16].

In terms of chromosomal distribution, our analysis uncovered intriguing chromosomal patterns for eccDNA origins. A notable role of chromosome 12 consistently featured prominently in both groups, potentially linked to its known susceptibility to chromothripsis, a major eccDNA biogenesis mechanism, suggesting a potential link between chromosome 12 vulnerability and eccDNA generation [32]. In addition, tumor-derived eccDNAs exhibited a slightly higher prevalence of being derived from multiple chromosomal fragments, indicating a considerable role of genomic rearrangements in eccDNA generation [12,29].

Identified eccDNAs in both tumor and non-tumor cell lines displayed a higher prevalence of intronic sequences compared to CpG islands, exons, 3’UTRs, and 5’UTRs, aligning with previous research [9,14,15,29]. Additionally, regardless of sample type, eccDNAs were enriched for transposable elements (TEs) and non-coding repeat regions. TEs, repetitive sequences like RNA and DNA transposons, play diverse roles in humans, including maintaining genomic stability, promoting rearrangements, and regulating gene expression [33]. However, uncontrolled TE activity is implicated in various diseases and cancers [34,35]. The detection of TEs in eccDNAs suggests potential mechanisms for their formation and hints at the regulatory roles of non-coding sequences within these circles. Further investigation is warranted to understand the functional roles and molecular mechanisms of TEs and non-coding regions within eccDNAs in the context of tumorigenesis. Regarding insertion and deletion patterns, we observed a lower insertion frequency in tumor samples compared to non-tumor samples.

Moreover, we investigated potential regulatory elements harbored by CCA-derived sequences. The identification of a 20 bp motif in eccDNA sequences suggests its potential involvement in tumorigenesis. The most significantly matched motif similarity to the ZNF560 motif hints at the possible involvement of C2H2-zinc finger factors and TEAD domain transcription factors in eccDNA formation or regulation. The exact roles of ZNF560 in cancer is still unclear, but it has been related to poor prognosis in patients with acute myeloid leukemia [36]. Further investigation is warranted to explore the potential regulatory role for the regulatory elements; ZNF560 and TEAD domain factors motif harbored by eccDNAs in cholangiocarcinogenesis.

In pathway network analysis, we observed that genes of the identified eccDNAs were enriched in some neurological pathways and pathways driven by families of RUNX transcription factors. We explored each unique pathway in which CCA-derived genes were involved and found that the role of GRB2 in signalling might be pivotally contributing to the carcinogenesis; leading to stimulation of Ras/BRAF pathway [37]. Moreover, RUNX-associated genes formed a cluster of connected networks, sharing common genes, suggesting that the biogenesis of the CCA-related eccDNAs might be originating from these gene locations.

We observed a positive trend between read depth coverage in the CCA cell line for key CCA-related genes, as well as distinct depth and coverage of oncogenes in CCA-derived eccDNAs. BRAF and KRAS, frequently mutated in CCA, displayed higher coverage in the KKU213A tumor cell line. Prevalence of BRAF mutation in CCA is increasing and gaining prominence [38]. BRAF, a downstream effector of KRAS in the RAS/RAF pathway, exhibited considerably higher read depth in our CCA cell line, KKU213A, compared to non-tumor one, MNNK1. However, individual replicates displayed distinct oncogene profiles, highlighting the heterogeneity of eccDNA composition, and the potential of cross interactions. In the CCA samples, replicate 1: BRAF, GNB1, ZYG11B, CYP4X1 (P450 family), and OSBPL9 were found on the eccDNAs. Replicate 2: FGFR2, KRAS, BRAF, NF1, ZYG11B, ARID1B, MAPK1 (ERK2), KMT2C, CD44, and OSBP2 were identified. Replicate 3: DOCK1, CCP110, SSBP3, ALG6, the stress sensor gene PEX5, and key transcription factors PRRX1 and TTF2 were detected on eccDNAs. Moreover, the choice of enzyme treatment can impact eccDNA detection and read depth. The observed differences in eccDNA read depth suggest that enzyme specificity influences the efficiency of eccDNA enrichment. While both approaches successfully captured eccDNA, their unique cutting mechanisms may result in differential read depth across oncogenes. Future studies should optimize enzyme selection to reduce potential biases and enhance the accuracy of eccDNA characterization.These findings suggest potential links between eccDNA enrichment and oncogene amplification in CCA. However, this higher coverage exhibited diverse patterns within characterized eccDNAs, and was not always observed in all replicates, consistent with previously reported heterogeneity in eccDNA origins and structures [25]. The observed diversity might be attributed to (i) inherent variation in eccDNA composition across samples [25], and (ii) potential technical biases during amplification, where, for example, the phi29 polymerase enzyme might show preference for certain eccDNAs in specific samples. These findings reinforce the complexity of eccDNA landscapes and underscore the need for further exploration of their roles in cancer development. Moreover, some studies have shown that eccDNA content in patient-derived cancer cell lines could differ from that of the original tissues [39], as eccDNAs may exhibit a tendency to reintegrate into the main chromosomes after prolonged *in vitro* culture [40]. With this in mind, our study on cell lines may represent a partial subset of relevant eccDNAs for CCA. Yet, it provides a promising and important foundation for future studies where valuable patients specimen will be investigated.

## Conclusion

Our study reveals the complex and diverse landscape of eccDNAs in CCA, offering several key insights. We identified replicate-specific oncogene characteristics in cancer-derived eccDNAs, underscoring their heterogeneity. The composition of genic and non-genic regions in the identified eccDNAs reinforce the conclusions drawn from previous studies while also revealing a unique 20 bp motif (CCCAGGCTGGAGTGCAGTGG) exclusive to cancer-derived eccDNAs. Notably, we observed a potential link between higher read depth coverage in eccDNAs that contained key genes in the RAS/BRAF pathway. These findings collectively highlight the intricate nature of eccDNA landscapes in CCA and their potential role in cancer pathogenesis. Importantly, it is setting the stage for further research using patient specimens combined with clinical intervention to fully elucidate their clinical and biological significance.

## Supporting information

S1 Table Run summary report. (DOCX)

S2 Table qPCR results of all samples. (DOCX)

S3 Table Chromosomal distribution of eccDNAs after normalized with respective chromosomal length. (DOCX)

S1 Fig GC content analysis and eccDNA unique count. (DOCX)

S2 Fig Gel electrophoresis result of PCR products and coverage visualized on IGV. (DOCX)

S3 Fig Flow chart of downstream data analysis. (DOCX)
