# Supplementary material for: Characterization and identification of extrachromosomal circular DNA in cholangiocarcinoma
Source: PLoS One. 2025 May 5;20(5):e0322173. doi: 10.1371/journal.pone.0322173 (PMC12052172; doi:10.1371/journal.pone.0322173)
Supplement: S1 Table — (DOCX) [file pone.0322173.s001.docx]

## S1 Table. Run summary report.

| **Sample name** | **Number of reads** | **Mean read length** | **Mean read quality** | **N50** |
| --- | --- | --- | --- | --- |
| Cas9_KKU213A_R1 | 307174 | 2225 | 15.3 | 3165 |
| Cas9_KKU213A_R2 | 326501 | 2149 | 15.2 | 3082 |
| Cas9_KKU213A_R3 | 291789 | 2622 | 15.1 | 3845 |
| MssI_KKU213A_R1 | 216327 | 2290 | 15 | 3370 |
| MssI_KKU213A_R2 | 209084 | 2311 | 15.2 | 3385 |
| MssI_KKU213A_R3 | 85523 | 2520 | 15.2 | 3683 |
| Cas9_MMNK1_R1 | 278151 | 2373 | 15.2 | 3531 |
| Cas9_MMNK1_R2 | 266726 | 2405 | 15.5 | 3457 |
| Cas9_MMNK1_R3 | 358073 | 2189 | 15.5 | 3185 |
| MssI_MMNK1_R1 | 175177 | 2473 | 15.1 | 3807 |
| MssI_MMNK1_R2 | 181329 | 2536 | 15.3 | 3783 |
| MssI_MMNK1_R3 | 366877 | 2294 | 15.5 | 3348 |
