# Supplementary material for: Characterization and identification of extrachromosomal circular DNA in cholangiocarcinoma
Source: PLoS One. 2025 May 5;20(5):e0322173. doi: 10.1371/journal.pone.0322173 (PMC12052172; doi:10.1371/journal.pone.0322173)
Supplement: S2 Table — (DOCX) [file pone.0322173.s002.docx]

## S2 Table. qPCR results of all samples.

| **Sample Name** | **HBB** | **MT-CO1** | **MT-ND1** | **MT-ND5** |
| --- | --- | --- | --- | --- |
| **KKU213A, Replicate I** | | | | |
| NC without primer | Undetermined | | | |
| NC (no gDNA) | 36.97 | 33.59 | 32.95 | 33.33 |
| PC | 27.86 | 22.67 | 18.82 | 21.95 |
| MssI | Undetermined | 35.05 | Undetermined | 33.50 |
| CRISPR | Undetermined | Undetermined | 35.46 | 37.06 |
| **KKU213A, Replicate II** | | | | |
| NC (no gDNA) | Undetermined | Undetermined | 37.96 | 29.91 |
| PC | 27.31 | 19.03 | 18.37 | 16.63 |
| MssI | Undetermined | 25.41 | 25.40 | 22.14 |
| CRISPR | 37.62 | 25.13 | 28.94 | 26.01 |
| **KKU213A, Replicate III** | | | | |
| NC (no gDNA) | 38.60 | 38.08 | 37.77 | 36.34 |
| PC | 26.278 | 19.17 | 19.77 | 19.16 |
| MssI | 38.126 | 26.90 | 31.29 | 25.37 |
| CRISPR | 39.06 | 24.04 | 28.8 | 25.59 |
| **MMNK-1, Replicate I** | | | | |
| NC without primer | Undetermined | | | |
| NC (no gDNA) | Undetermined | Undetermined | 37.96 | 36.91 |
| PC | 27.42 | 18.85 | 19.90 | 18.29 |
| MssI | 37.90 | 26.62 | 25.90 | 21.83 |
| CRISPR | 38.79 | 25.87 | 24.87 | 29.58 |
| **MMNK-1, Replicate II** | | | | |
| NC (no gDNA) | 38.60 | 38.08 | 37.77 | 36.34 |
| PC | 27.99 | 19.60 | 20.39 | 19.62 |
| MssI | 37.20 | 25.57 | 31.49 | 28.27 |
| CRISPR | 38.39 | 24.13 | 27.66 | 23.97 |
| **MMNK-1, Replicate III** | | | | |
| NC (no gDNA) | 39.60 | 38.30 | 37.17 | 37.34 |
| PC | 30.10 | 19.86 | 20.89 | 20.13 |
| MssI | 37.75 | 27.02 | 34.27 | 28.42 |
| CRISPR | 38.72 | 22.46 | 27.55 | 24.88 |
| The qPCR reactions were run with 40 cycles. NC = Negative control, PC = Positive control (Post extracted DNA for each replicate). | | | | |
