# Supplementary material for: Characterization and identification of extrachromosomal circular DNA in cholangiocarcinoma
Source: PLoS One. 2025 May 5;20(5):e0322173. doi: 10.1371/journal.pone.0322173 (PMC12052172; doi:10.1371/journal.pone.0322173)
Supplement: S3 Table — (DOCX) [file pone.0322173.s003.docx]

## S3 Table. Chromosomal distribution of eccDNAs after normalized with respective chromosomal length

| **Chr No.** | **MssI_KKU213A** | **Cas9_KKU213A** | **MssI_MMNK1** | **Cas9_MMNK1** |
| --- | --- | --- | --- | --- |
| chr 1 | 1.77E-06 | 1.61E-06 | 1.59E-06 | 1.73E-06 |
| chr 2 | 1.42E-06 | 1.45E-06 | 1.76E-06 | 1.87E-06 |
| chr 3 | 2.17E-06 | 2.25E-06 | 1.74E-06 | 1.83E-06 |
| chr 4 | 1.21E-06 | 1.34E-06 | 1.58E-06 | 1.71E-06 |
| chr 5 | 1.74E-06 | 1.37E-06 | 1.74E-06 | 1.94E-06 |
| chr 6 | 1.89E-06 | 1.79E-06 | 2.15E-06 | 2.11E-06 |
| chr 7 | 1.47E-06 | 1.49E-06 | 2.04E-06 | 2.29E-06 |
| chr 8 | 1.87E-06 | 1.60E-06 | 1.47E-06 | 1.43E-06 |
| chr 9 | 1.63E-06 | 1.48E-06 | 1.30E-06 | 1.32E-06 |
| chr 10 | 1.63E-06 | 1.45E-06 | 1.79E-06 | 1.80E-06 |
| chr 11 | 1.60E-06 | 1.51E-06 | 1.81E-06 | 1.63E-06 |
| chr 12 | 2.10E-06 | 1.90E-06 | 1.80E-06 | 1.88E-06 |
| chr 13 | 1.67E-06 | 1.58E-06 | 1.57E-06 | 1.60E-06 |
| chr 14 | 1.44E-06 | 1.53E-06 | 1.33E-06 | 1.43E-06 |
| chr 15 | 1.23E-06 | 1.31E-06 | 9.02E-07 | 8.04E-07 |
| chr 16 | 1.33E-06 | 1.26E-06 | 1.16E-06 | 1.03E-06 |
| chr 17 | 1.61E-06 | 1.40E-06 | 1.42E-06 | 1.38E-06 |
| chr 18 | 1.24E-06 | 1.15E-06 | 1.72E-06 | 1.82E-06 |
| chr 19 | 1.69E-06 | 1.29E-06 | 5.64E-07 | 6.48E-07 |
| chr 20 | 2.28E-06 | 1.79E-06 | 1.72E-06 | 2.00E-06 |
| chr 21 | 9.20E-07 | 1.07E-06 | 1.52E-06 | 1.18E-06 |
| chr 22 | 8.07E-07 | 7.28E-07 | 6.49E-07 | 6.88E-07 |
| chr X | 8.46E-07 | 9.09E-07 | 1.00E-06 | 1.05E-06 |
| chr Y | 0.00E+00 | 5.24E-08 | 1.75E-08 | 1.75E-08 |
| chr M | 0.00E+00 | 1.81E-04 | 0.00E+00 | 6.04E-05 |
