# Supplementary material for: Characterization and identification of extrachromosomal circular DNA in cholangiocarcinoma
Source: PLoS One. 2025 May 5;20(5):e0322173. doi: 10.1371/journal.pone.0322173 (PMC12052172; doi:10.1371/journal.pone.0322173)
Supplement: S1 Fig — (DOCX) [file pone.0322173.s004.docx]

##
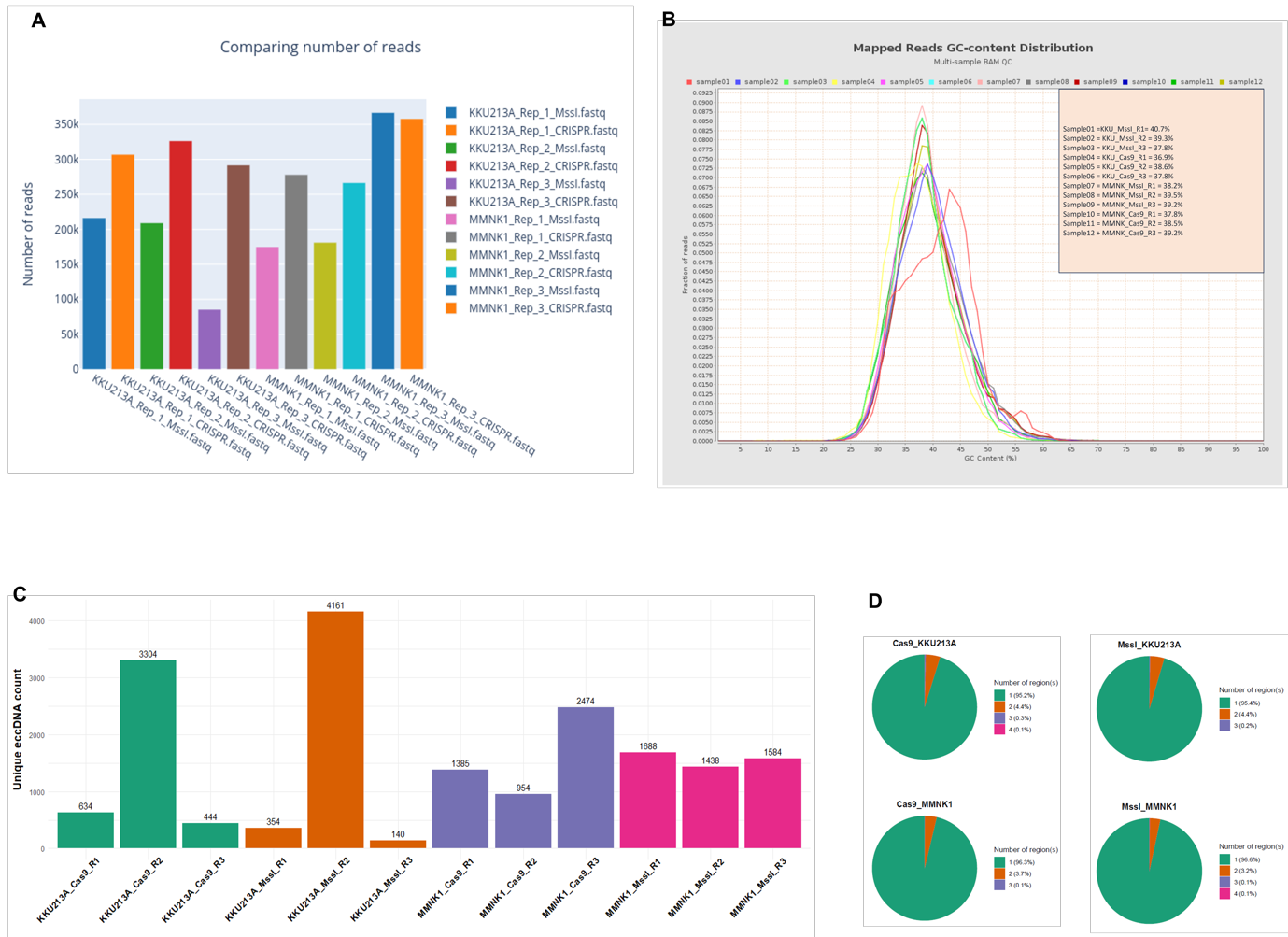


### S1 Fig. GC content analysis and eccDNA unique count.

**(A)** Comparison of number of reads for all samples

**(B)** GC content distribution of mapped sequences

**(C)** Total number of unique eccDNA

**(D)** Single vs. multiple fragment origin of eccDNAs
