# Supplementary material for: Characterization and identification of extrachromosomal circular DNA in cholangiocarcinoma
Source: PLoS One. 2025 May 5;20(5):e0322173. doi: 10.1371/journal.pone.0322173 (PMC12052172; doi:10.1371/journal.pone.0322173)
Supplement: S2 Fig — (DOCX) [file pone.0322173.s005.docx]

| 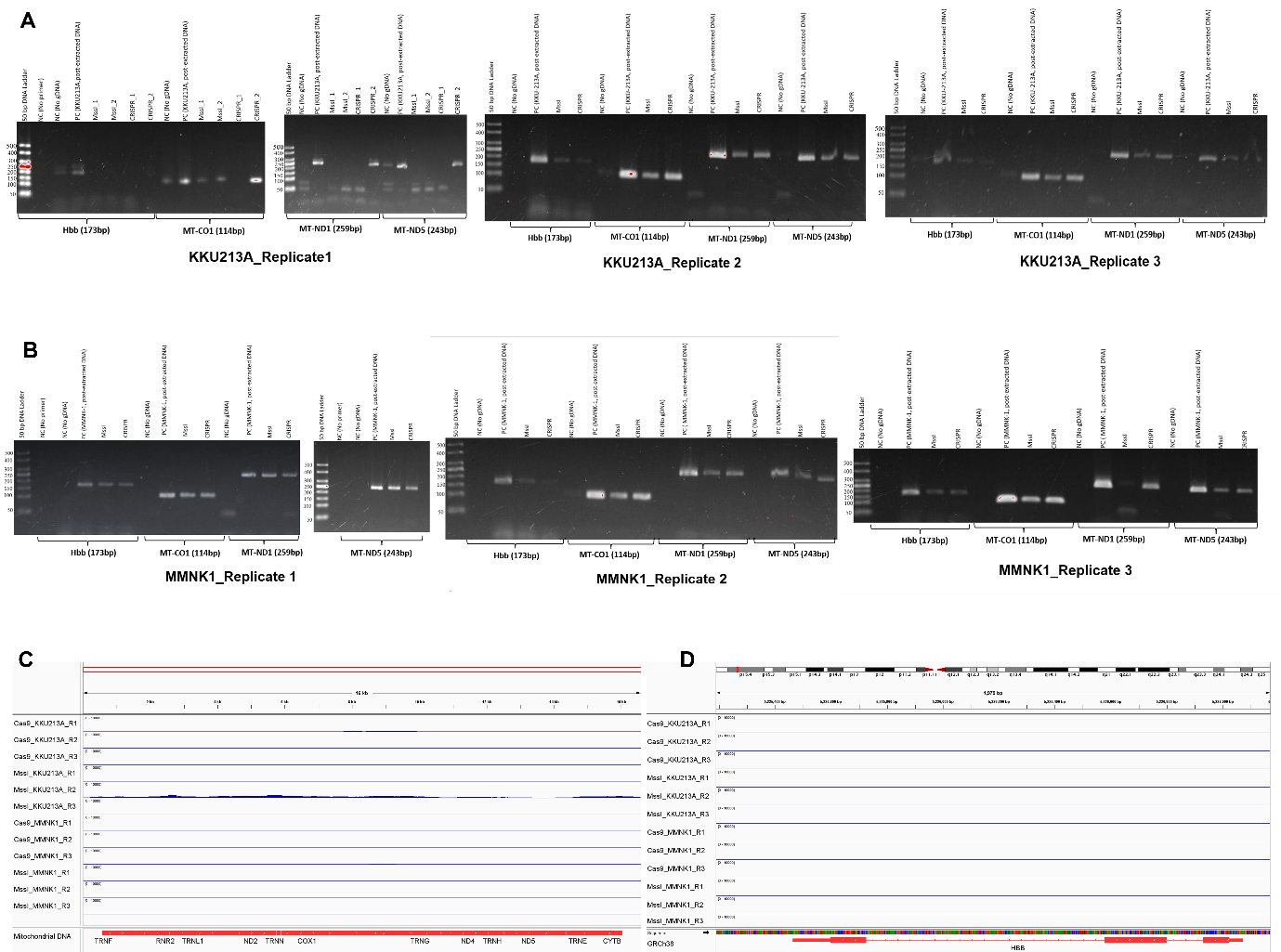 |
| --- |

### **S2 Fig. Gel electrophoresis result of qPCR products and coverage visualised on IGV**.

1. KKU213A cell line
2. MMNK1 cell line
3. Mitochondrial DNA visualized by IGV
4. Genomic DNA removal visualized by IGV using the HBB gene as an indicator
