# Supplementary material for: Characterization and identification of extrachromosomal circular DNA in cholangiocarcinoma
Source: PLoS One. 2025 May 5;20(5):e0322173. doi: 10.1371/journal.pone.0322173 (PMC12052172; doi:10.1371/journal.pone.0322173)
Supplement: S3 Fig — (DOCX) [file pone.0322173.s006.docx]

| **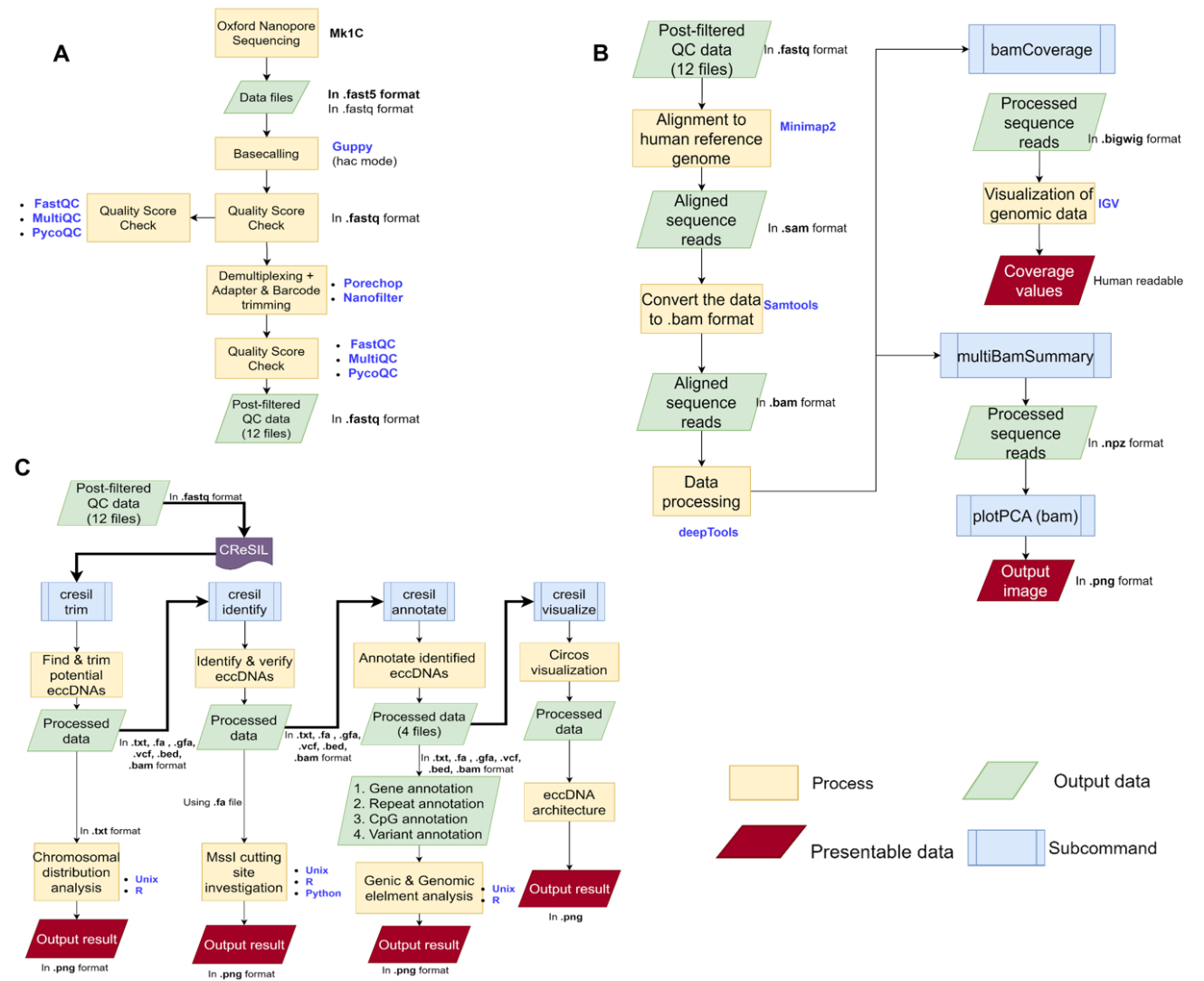** |
| --- |
|  |
| **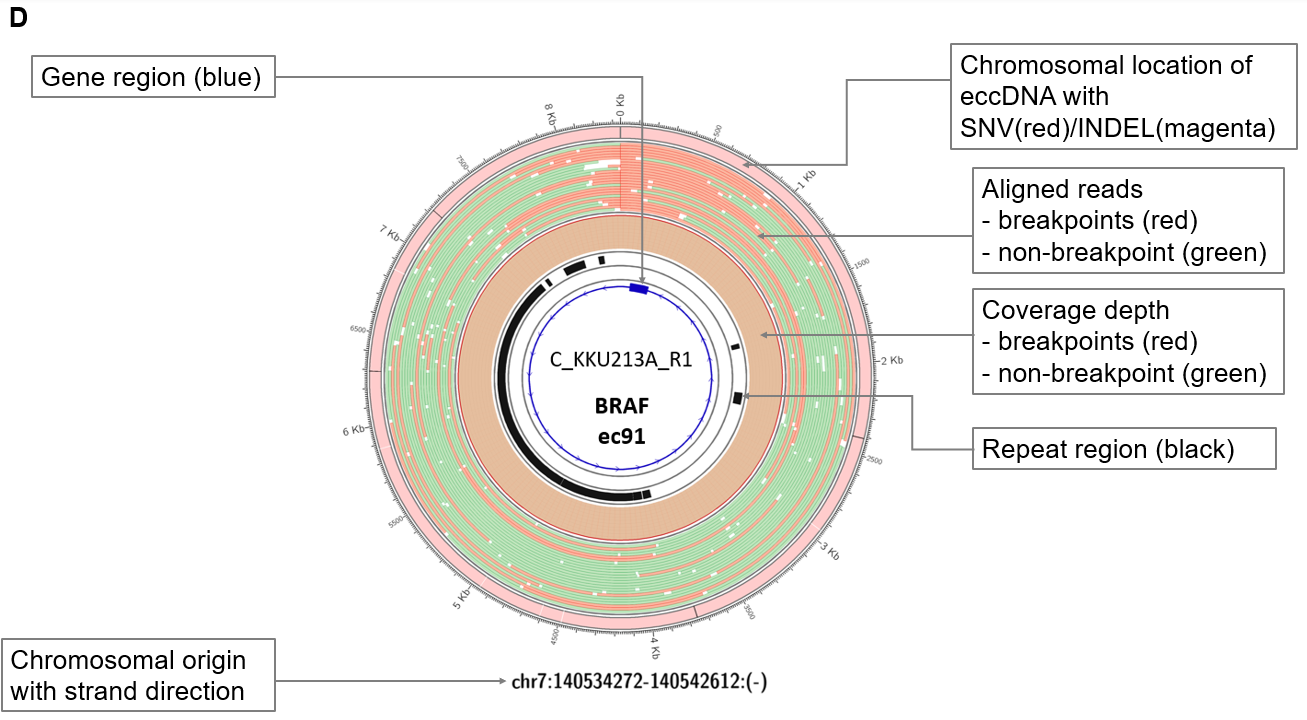** |

## **S3 Fig. Flow chart of downstream data analysis**.

1. Schematic chart of data analysis using data received from sequencer, performed base-calling for the sequencing and the output data was used to trim adapter and barcode
2. Flowchart of the data analysis using genome mapping data, trimmed and post-filtered sequences were mapped to human reference genome and the resulting files were used for further analysis
3. General workflow of identification and characterization of eccDNA using CReSIL pipeline, including the number of unique eccDNAs, the trimmed post-filtered QC passed reads in .fastq format were parsed to CReSIL pipeline which outputs the assembled sequences
4. Circos visualization to present the eccDNA architecture. To visualize eccDNA architecture in circos, CReSIL generated an input file of selected eccDNA. The circos illustration contains necessary information such as the chromosomal origin and coverage sketch of eccDNA.
